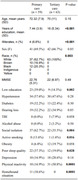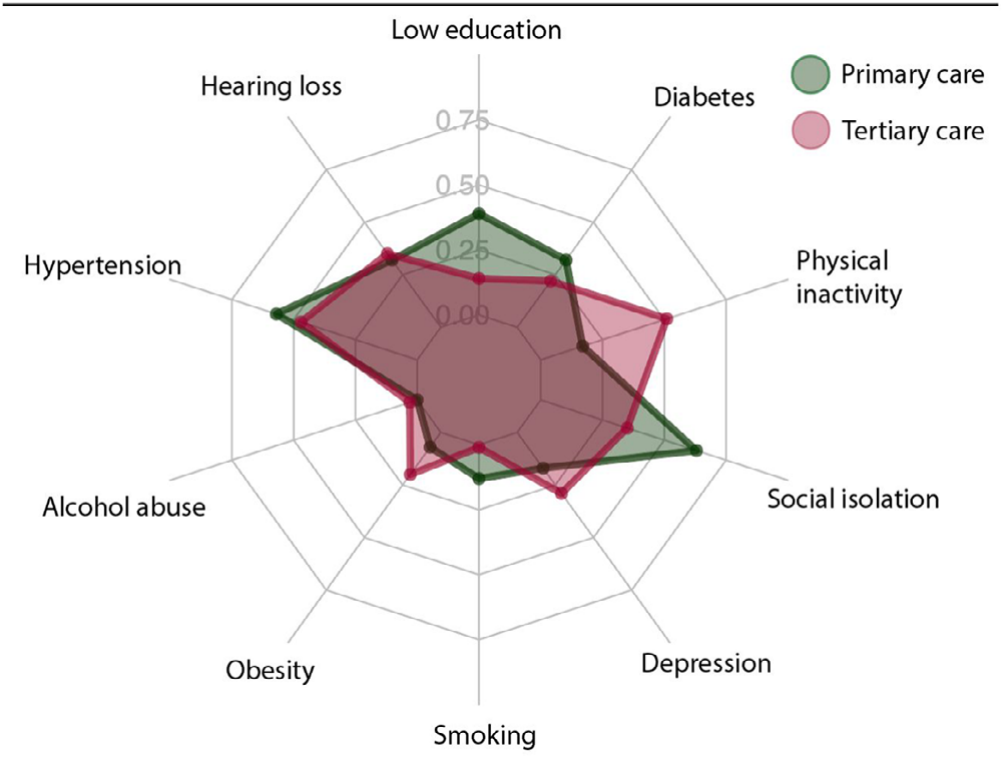# Frequency of modifiable risk factors in primary care compared with tertiary care in Brazil

**DOI:** 10.1002/alz.092547

**Published:** 2025-01-09

**Authors:** Núbia Alencar de Freitas, Wyllians Vendramini Borelli, Pedro Rosa‐Neto, Raphael Machado Castilhos, Sheila O Martins, Vivian Silveira Vasques, Giovanna Carello‐Collar, Brunna Jaeger Tello, Eduardo R. Zimmer, Jeanette Inglez‐de‐Souza Farina, Mariana Dagnino Araujo, Alberto Luiz Grigoli Maia, Anelise Fuhr

**Affiliations:** ^1^ Universidade Federal do Rio Grande do Sul, Porto Alegre Brazil; ^2^ Federal University of Rio Grande do Sul, Porto Alegre, Rio Grande do Sul Brazil; ^3^ Memory Center, Hospital Moinhos de Vento, Porto Alegre, RS Brazil; ^4^ McGill Centre for Studies in Aging/Translational Neuroimaging Laboratory, Montreal, QC Canada; ^5^ Memory Center, Hospital Moinhos de Vento, Porto Alegre, RS, Brazil Brazil; ^6^ Universidade Federal do Rio Grande do Sul, Porto Alegre, Rio Grande do Sul Brazil; ^7^ Universidade Federal do Rio Grande do Sul, Porto Alegre, RS Brazil

## Abstract

**Background:**

The Better at Home Program (BHP) is a Brazilian government initiative to provide dehospitalization through homecare health access in primary care. The profile of modifiable risk factors of dementia in this vulnerable population may provide targets for public health policies, as it represents the majority of the Brazilian population. In this study, we aimed at investigating the profile of modifiable risk factors in primary care compared with a tertiary care clinic in Brazil.

**Method:**

Community‐dwelling patients attending the BHP and from tertiary memory clinics were invited to participate. We collected demographic variables, the profile of modifiable risk factors of dementia, neuropsychological battery and blood sampling of both primary and tertiary care settings. Frequency of risk factors were compared with chi‐squared tests and quantitative demographic variables were compared with parametric tests.

**Results:**

A total of 59 patients were included in primary care (mean 72.3±7.9 years of age, 62.7% females) and 63 in tertiary care setting (mean 70±11 years, 66.6% females). Primary care patients were significantly less educated compared with tertiary centers (7.44±3.9 vs. 16±5.34), and more racially diverse (Table), with similar MMSE scores (22.76±5.31 vs. 22±6.97). The profile of frequency of modifiable risk factors of dementia was distinct according to the level of care (Figure). Specifically, individuals from primary care presented increased frequency of low education (23 vs. 3, p = 0.03), social isolation (37 vs. 22, p = 0.007), physical inactivity (31 vs 10, p = 0.0001) and smoking. Furthermore, people in homebound situations were identified only in the primary care group (11 vs. 0, p<0.001).

**Conclusion:**

Patients from primary care services present a distinct profile of modifiable risk factors of dementia when compared with tertiary care. They presented increased frequency of low education, social isolation, physical inactivity, and smoking. Public policies targeting primary care populations in LMIC may address risk factors differently.